# Characteristics and candidate genes associated with excellent stalk strength in maize (*Zea mays* L.)

**DOI:** 10.3389/fpls.2022.957566

**Published:** 2022-07-28

**Authors:** Xiaqing Wang, Yining Chen, Xuan Sun, Jinghuan Li, Ruyang Zhang, Yanyan Jiao, Ronghuan Wang, Wei Song, Jiuran Zhao

**Affiliations:** Beijing Key Laboratory of Maize DNA Fingerprinting and Molecular Breeding, Maize Research Institute, Beijing Academy of Agriculture and Forestry Sciences, Beijing, China

**Keywords:** maize, stalk strength, cell wall, phenylpropanoid, cellulose, hemicellulose, differentially expressed genes

## Abstract

Lodging is a major problem in maize production, which seriously affects yield and hinders mechanized harvesting. Improving stalk strength is an effective way to improve lodging. The maize inbred line Jing2416 (J2416) was an elite germplasm in maize breeding which had strong stalk mechanical strength. To explore the characteristics its stalk strength, we conducted physiological, metabolic and transcriptomic analyses of J2416 and its parents Jing24 (J24) and 5237. At the kernel dent stage, the stalk rind penetrometer strength of J2416 was significantly higher than those of its two parents in multiple environments. The rind thickness, sclerenchyma tissue thickness, and cellulose, hemicellulose, and lignin contents of J2416 were significantly higher than those of its parents. Based on the significant differences between J2416 and 5237, we detected metabolites and gene transcripts showing differences in abundance between these two materials. A total of 212 (68.60%) metabolites and 2287 (43.34%) genes were up-regulated in J2416 compared with 5237. The phenylpropanoid and glycan synthesis/metabolism pathways were enriched in metabolites and genes that were up-regulated in J2416. Twenty-eight of the up-regulated genes in J2416 were involved in lignin, cellulose, and hemicellulose synthesis pathways. These analyses have revealed important physiological characteristics and candidate genes that will be useful for research and breeding of inbred lines with excellent stalk strength.

## Introduction

Lodging, the breaking of the stalk, is a major problem in the production of maize (*Zea mays* L.). It causes maize plants to fall over, thereby affecting photosynthesis and development, and rendering the plants more vulnerable to damage from pests and diseases (Shah et al., 2017). Lodging can occur at different stages of development, but it causes the most serious problems when it occurs at the grain development stage. At this stage, lodging hinders the transport of photosynthates to the grain, thereby negatively affecting the maize yield (Wen et al., 2019; Zhao and Zhou, 2022). Stalk lodging also affects mechanized harvesting. It has been reported that when the lodging rate increases by 1%, the ear drop rate increases by 0.15% (Xue et al., 2018). With the development of mechanization for modern maize cultivation, the demand for maize varieties that retain upright stalks during harvesting is increasing, and consequently, there is a need for new varieties with strong stalk strength. Therefore, improving stalk strength is of great significance for maize lodging resistance, stabilizing maize production, and accelerating the modernization of maize production.

There are many indicators of stalk strength, such as rind penetrometer strength, stalk bending strength, and stalk bending angle. Rind penetrometer strength has been widely used by many researchers. Because it easy to measure, this trait can be measured multiple times in different positions on the same stalk to obtain more accurate data (Li et al., 2014; Wang et al., 2019). Stalk strength and the position of fracturing differ among developmental stages. At the early stage of tasseling, the stalk is easily broken at 1 cm above the first node above the ear (Wang et al., 2019). At the grain filling stage, the maize stalk is prone to breaking at the internode of the third node at the base. This may be because the height of the center of gravity moves upwards during growth and some of the photosynthates that stabilize the stalk are transported to the grain (Xu et al., 2017; Stein and Granot, 2019; Shah et al., 2021).

Stalk strength is affected by several physiological traits, including stalk shape, stalk anatomical characteristics, and cell wall components, i.e., the types and contents of various structural compounds and metabolites (Shah et al., 2017). Varieties with a short and thick internode at the base are more resistant to lodging (Kotake et al., 2011; Okuno et al., 2014). The internal structure of the stalk determines its external macroscopic morphology and mechanical properties. The anatomical features of the maize stalk include the epidermis, sclerenchyma cells, parenchyma cells, and vascular bundles. In recent years, X-ray microcomputed tomography technology has allowed for the observation of the total number of vascular bundles in a cross-section of the entire stalk. This technology has greatly facilitated the observation of the number and shape of vascular bundles (Du et al., 2016). Studies have shown that stalk strength is positively correlated with the thickness of the rind and sclerenchyma tissue, as well as the number and density of vascular bundles (Kong et al., 2013; Xue et al., 2016; Wang et al., 2019; Guo et al., 2021). The stalk consists of many chemical components, such as cellulose, hemicellulose, lignin, pectin, and starch. The most abundant components are cellulose, hemicellulose, and lignin. Cellulose is the largest polymer in plant cell walls and provides mechanical support for cells. Hemicellulose and cellulose together form a network; and lignin exists in a highly complexed state in thickened secondary cell walls, providing mechanical support (Qiu and Hu, 2013; Yoon et al., 2015; Deshavath et al., 2017). Cellulose, hemicellulose, and lignin contents are all positively related to stalk strength (Jiao et al., 2019; Wang et al., 2020; Zhang et al., 2020). With the development of phenomics technology, more novel phenotypes have been revealed. Metabolite analysis has been gradually applied in crop genetics research because of their diversity and extremely sensitive response to changes in gene expression (Wen et al., 2016). However, few studies have explored the roles of particular metabolites in stalk development and stalk strength.

The germplasm of the Huang-gai group is an extremely important heterotic group in maize breeding. Because of its excellent characteristics, this group is often used for gene mining and breeding of new maize varieties. The inbred lines J24 and 5237 are elite lines derived from the Huang-gai germplasm. Some popular maize hybrids such as Jingdan 28 and Yedan 22 have been bred from J24 and 5237, respectively (Chen et al., 2009). Recurrent selection breeding from the hybrids of these two inbred lines has resulted in some excellent inbred lines, among which J2416 ([(J24 × 5237) × J24]) is the most successful. To date, more than 21 commercial maize hybrids have been bred from J2416, and more than four hybrids are cultivated across a planting area of more than 67,000 ha (Zhao et al., 2020a). Eighteen varieties bred from J2416 have particularly strong stalk strength (unpublished). Therefore, J2416 has become a backbone inbred line for research and breeding.

To explore the mechanism of the excellent stalk strength of J2416, we analyzed the anatomical and biochemical traits of its stalk, and detected metabolites that differed significantly in abundance compared with its parents (J24 and 5237). Genes that were up-regulated in J2416 and closely related to stalk strength traits were identified. Our results reveal which characteristics and candidate genes are related to the strong stalk phenotype, and provide a reference for developing maize varieties with high-quality stalks.

## Materials and methods

### Plant materials

**FIGURE 1:**
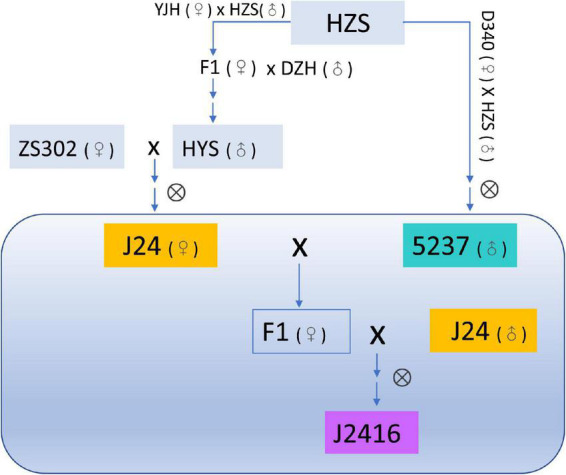
Pedigree of J2416 and its parents J24 and 5237.

The maizel inbred line J2416 and its parents J24 and 5237 were obtained from the Maize Research Institute, Beijing Academy of Agriculture and Forestry Sciences, Beijing, China. All the three materials belong to the germplasm of the heterotic group of Huang-gai. J24 was bred from a hybrid of the early maturing line 302 (ZS302) and Huangyesi (HYS) (Figure 1). 5237 was bred from a hybrid of Huangzaosi (HZS) and Dan340. J2416 was bred from the backcross population of J24 and 5237 [(J24 × 5237) × J24] (Zhao et al., 2020a; Figure 1).

### Field trial

The three inbred lines J2416, J24, and 5237 were planted at Sanya, Hainan (HN, 108.56°E, 18.09°N) in winter 2020; Tongzhou, Beijing (TZ, 116.65°E, 39.92°N), and Changping Beijing (CP, 119.39°E, 40.17°N) in summer 2021. In all environments, each inbred line was planted in five rows, with 20 plants per row (plant spacing of 25 cm; row spacing of 60 cm). Irrigation and pest control were consistent with local agronomic practices throughout the growth and development of maize.

### Stalk mechanical strength measurements

To study the stalk strength of these materials at different stages after silking, stalk strength was measured for 5237, J2416, and J24 at the kernel dent stage, corresponding to about 31 to 33 days after the R1 stage (silks extending outside the husk leaves) of maize, respectively (Ciampitti et al., 2011). During this period, kernels are “dented” at the tip because of declining moisture content and increasing starch content, and husk leaves are fading to a pale green with browning on the edges (Ciampitti et al., 2011). At this stage, the stalks of the three materials were still standing upright and were not dry. Rind penetrometer strength was measured at the third internode with 9–60 uniform plants using a YYD-1 instrument (Zhejiang Top Cloud-Agri Technology Co., Ltd., Zhejiang, China) (Wang et al., 2019).

### Stalk anatomical characteristics

To investigate anatomic traits, twenty 200-μm hand-cut sections were prepared from the third stalk at the base of the stalk of the three materials. The materials were collected at 30DAS_21TZ (30 days after silking in Tongzhou in summer 2021). The sections were stained with 2% phloroglucinol dye for 30–60 s (Sindhu et al., 2007). The anatomical characteristics were observed under an Olympus DP80 compound microscope (Olympus Corp., Shinjuku, Japan). Images were analyzed with Image-Pro Plus 6.0 to measure the thickness of the stalk rind and sclerenchyma. The results were statistically analyzed using the R software package.

### Detection of stalk cell wall components

After measuring the stalk strength of each material, stalks were collected with three to five replicates per material, with five plants per replicate on 30DAS_21TZ. The material was naturally air-dried, and then dried at 40°C before grinding and sieving. Samples were passed through 40 to 80 mesh sieves (0.425 mm–0.180 mm fineness) before extracting structural carbohydrates and lignin using a two-step sulfuric acid hydrolysis process. The contents of cellulose, hemicellulose and lignin were determined using a high-performance liquid chromatography system (HPLC, 1260 series, Agilent Technologies, Santa Clara, CA, United States) (Sluiter et al., 2010).

### Metabolite detection and analysis

For each material, stalk samples were taken at 1 cm above the third stalk node, and were stored at −80°C. Three replicates of each material, with each replicate consisting of three mixed samples, were collected on 15DAS_21TZ (15 days after silking in 2021 at Tongzhou base). The extraction, detection and analysis methods of metabolites were as follows.

Extraction process of metabolite. 50 mg of the sample powder was added to 1000 μL of the extraction solution containing the internal standard (volume ratio of methanol to acetonitrile to water as 2:2:1, internal standard concentration as 2 mg/L), and vortexed for 30 s. The samples were grinded for 10 min, and then used for ultrasonic extraction for 10 min at low temperature. Next, the samples were kept in −20°C for 1 h. The supernatant was dried in a vacuum concentrator after centrifugation at 4°C for 15 min (12000 rpm). The extraction solution (volume ratio of acetonitrile to water as 1:1) was added to the dried substance to reconstitute. The solution was vortexed again for 30 s, and then used for ultrasonic extraction for 10 min at low temperature. The mixture was centrifuged at 12000 rpm for 15 min at low temperature, and the supernatant liquid was transferred into the injection vial.

Detection of metabolites. Liquid chromatography–tandem mass spectrometry (LC-MS/MS) system used for metabolites detection consisted of a Waters Acquity I-Class PLUS UHPLC coupled with a Waters Xevo G2-XS QTof high-resolution mass spectrometer (Song et al., 2016). The column used in this system is an Acquity UPLC HSS T3 column (100mm × 2.1 mm, 1.8 mm, Waters, Milford, MA, United States), with the injection volume of 1 μL. The mobile phases A consisted of 0.1% formic acid aqueous solution. The mobile phase B consisted of 0.1% formic acid acetonitrile. The gradient parameters were based on Song et al., 2016. The Waters Xevo G2-XS QTof high-resolution mass spectrometer can perform primary and secondary mass spectrometry data acquisition in MSE mode under the control of the acquisition software (MassLynx V4.2, Waters). Dual-channel data acquisition can be performed simultaneously for both low collision energy and high collision energy in each data acquisition cycle. The low collision energy was 2 V, and the high collision energy was 10∼40 V. The scanning frequency was 0.2 s for a mass spectrum. ESI ion source parameters were set as follows: capillary voltage as 2000 V (positive ion mode) or −1500 V (negative ion mode); cone voltage as 30 V; ion source temperature as 150°C; desolvation gas temperature as 500°C; backflush gas flow rate as 50 L/h; flow rate of desolventizing gas as 800 L/h.

Analysis of metabolites. The raw data collected by MassLynx V4.2 was processed by Progenesis QI (Waters, Milford, MA, United States) software for peak extraction, peak alignment and other data processing operations. Metabolites were identified based on METLIN database (Smith et al., 2005). The differentially accumulated metabolites were selected on the basis of the *P*-value of the Student’s *t*-test and the variable importance value (VIP) from the Orthogonal Projections to Latent Structures Discriminant Analysis (OPLS-DA) model. Metabolites with a *P*-value < 0.05 and VIP > 1 were considered to be differentially accumulated between the two compared materials (Wiklund et al., 2008). The function of the differentially accumulated metabolites was determined by searches at the KEGG database (Kanehisa et al., 2019). Enrichment and hierarchical clustering analyses were conducted using KOBAS2.0 software^1^ (Altschul et al., 1997; Xie et al., 2011).

### Transcriptome sequencing and expression data analyses

The samples used for RNA sequencing and analysis were the same as those used for metabolite detection. Total RNA was extracted using TRIZOL reagent (Invitrogen, Carlsbad, CA, United States). Sequencing libraries were generated using the NEBNext Ultra™ RNA Library Prep Kit for Illumina (NEB, Ipswich, MA, United States), and sequenced using an Illumina HiSeq™ 2000 system. The clean reads were mapped to the maize reference genome (B73 AGPv4) using Hisat2 software (Kim et al., 2019). Gene expression levels were calculated as fragments per kilobase of transcript per million fragments mapped (FPKM) values (Florea et al., 2013). Genes with an abs (log2(fold change)) > 1 and false discovery rate (FDR) < 0.01 were considered as differentially expressed genes (DEGs) (Anders and Huber, 2010). The function of DEGs was determined according to annotations at the KEGG database (Kanehisa et al., 2019).

### Real-time quantitative PCR

The RNA samples used for RT-qPCR were the same as those used for transcriptome sequencing. The cDNA synthesis was performed using cDNA synthesis kit (HiScript**III** 1st Strand cDNA Synthesis Kit, Vazyme Biotech Company, China), according to the manufacturer’s protocol. The primers used for RT-qPCR were synthesized at the Tanyibiotech Company. The PCR system consisted of cDNA (2.5 uL), 2x Taq Pro Universal SYBR qPCR Master Mix (Q172, vazyme, 10 μL), 10 μM F and R primers (0.4 μL each), and water (6.7 μL). The PCR program was set as 95°C, 30 s; 40 cycles (95°C, 10 s; and 60°C, 30 s). Melting curves were established for the PCR products at the end of the amplification reaction (95°C, 15 s; 60°C, 60 s; 95°C, 15 s) with slow heating from 60°C to 99°C (automatically ramped the temperature at a rate of 0.05°C/s). The procedure was carried out on Quant Studio 6 Flex Real-Time PCR System (Thermo Fisher Scientific ABI, United States). ZmActin1 was used as the internal control (Zhang et al., 2018). The expression level of target genes was calculated using the relative 2^–ΔΔ*Ct*^ method (Livak and Schmittgen, 2001). All primers used for RT–qPCR was listed in Supplementary Table 1.

## Results

### Variation in stalk strength at the kernel dent stage

**FIGURE 2:**
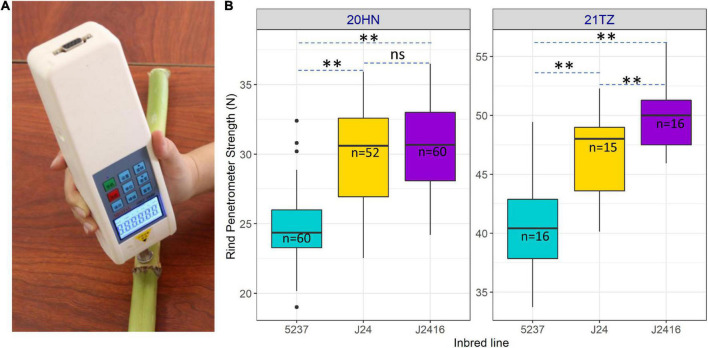
Rind penetrometer strength of J2416 and its two parents. **(A)** Measurement of rind penetrometer strength. **(B)** Rind penetrometer strength of three inbred lines in different locations. **Means the significant difference at 0.01 level. ns means no significant difference.

To compare stalk strength among J2416, J24 and 5237, we measured their rind penetrometer strength at the kernel dent stage in two different environments (Figure 2A). Although they were all 30 days after silking, the rind penetrometer strength of the same material in different environments was different. However, we detected significant differences in rind penetrometer strength among the three inbred lines across 22 degrees north latitude and 11 degrees east longitude, regardless of whether planting was in summer or winter. We found the rind penetrometer strength of J2416 was slightly higher than that of J24, but significantly higher than that of 5237 (Figure 2B).

### Variation of stalk anatomy traits and cell wall components

**FIGURE 3:**
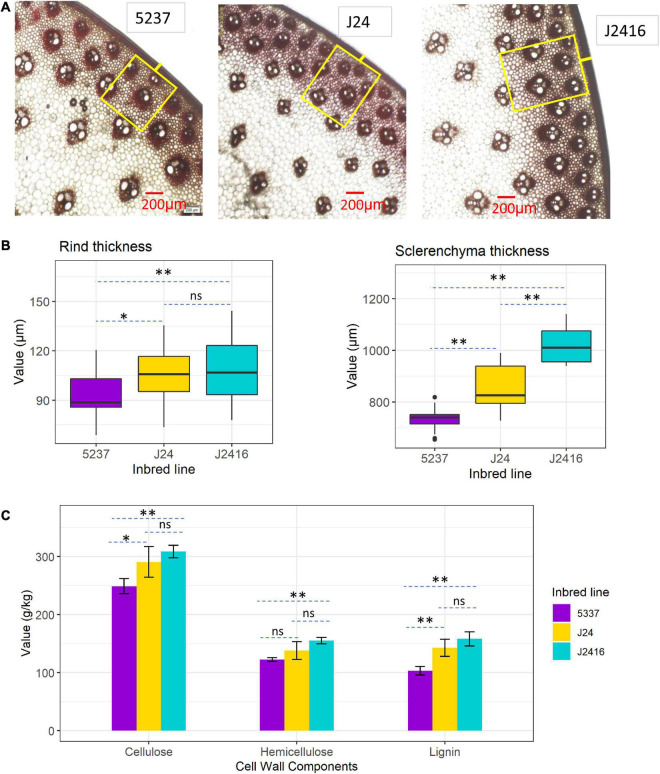
Stalk anatomical characteristics and cell wall components in J2416 and its two parents. **(A)** Anatomical characteristics observed in stalk cross sections (200 μm field) under a microscope. Yellow box represents sclerenchyma tissue; short yellow line in the middle of the box represents rind cells. **(B)** Rind thickness and sclerenchyma thickness in stalks of the three materials. **(C)** Contents of cell wall components in stalks of J2416 and its two parents. *, **Means the significant difference at 0.05 and 0.01 level, respectively. ns means no significant difference.

To understand the internal structure of the superior stalk-strength materials, we carried out anatomical observations of stalk cross-sections of the three materials collected at the kernel dent stage. Stalk sections of each material were stained with phloroglucinol, and then the internal structural characteristics of the stalk were observed under a microscope (Figure 3A). All the three materials had normal and similar organizational structure, with well-developed secondary cell walls (Figure 3A). The rind thickness of J2416 was significantly greater than that of 5237, but no significant difference with J24 (Figure 3B). The sclerenchyma thickness varied substantially among the three materials. The sclerenchyma thickness of J2416 was significantly greater than those of J24 and 5237. The sclerenchyma thickness of J24 was also significantly greater than that of 5237 (Figure 3B).

The contents of cellulose, hemicellulose and lignin in the stalk were determined by HPLC. All the contents of the three substances were significantly different between J2416 and 5237, but no significant difference between J2416 and J24 (Figure 3C). In addition, cellulose and lignin were also significantly different between J24 and 5237. The results of cell wall components together with stalk anatomic feature indicated that there were minor differences between J2416 and J24.

### Differentially accumulated metabolites in stalks

To explore the mechanisms of the differences in physiological characteristics of the stalks among the three inbred lines, we detected metabolites using LC-MS/MS technology. We detected 714 and 1082 metabolites in the positive and negative ion modes, respectively. Next, we divided the three materials into two comparison groups (group 1, J2416 vs. 5237; group 2, J2416 vs. J24) to detect differentially accumulated metabolites.

**FIGURE 4:**
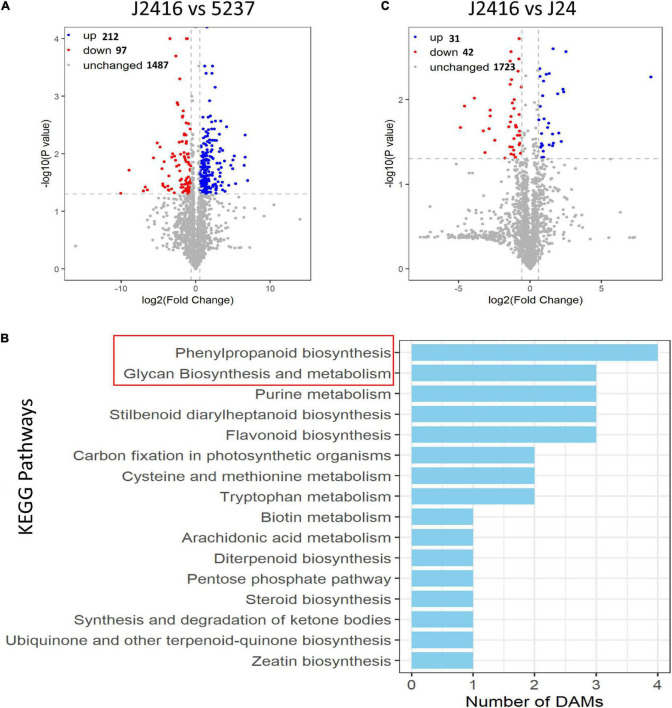
Differentially accumulated metabolites (DAMs). **(A)** Volcano plot of differentially accumulated metabolites between J2416 and 5237. Blue and red dots represent significantly up- and down-regulated metabolites, respectively, in J2416 compared with 5237. **(B)** KEGG pathways enriched with up-regulated metabolites in J2416. **(C) (A)** Volcano plot of differentially accumulated metabolites between J2416 and J24.

We detected 309 differentially accumulated metabolites between J2416 and 5237, consisting of 179 and 130 detected in the positive and negative ion modes, respectively (Figure 4A). In total, 212 metabolites (68.60%) were up-regulated in J2416. In order to obtain the specific biological functions and the metabolic networks of 212 metabolites, we used KOBAS2.0 software to annotate these metabolites into the KEGG database (Kanehisa et al., 2019). However, as the limitation of this database, only 30 metabolites were annotated into 16 KEGG pathways (Figure 4B). The number of up-regulated metabolites in each of these KEGG pathways varied from one to four. The phenylpropanoid pathway contained the largest number of differentially accumulated metabolites (four), followed by the glycan synthesis and metabolism pathways, flavonoid biosynthesis, purine metabolism, and stilbenoid diarylheptanoid biosynthesis pathways (three metabolites each). The other pathways contained one or two differentially accumulated metabolites.

Only 73 differentially accumulated metabolites were detected between J2416 and J24, consisting of 51 and 22 detected in the positive and negative ion modes, respectively. Of these, 31 were up-regulated in J2416, and six of those were in the polysaccharide biosynthesis and metabolism pathways (Figure 4C).

### Differentially expressed genes

According to the above phenotypes and metabolome analysis, we found there were few differences between J2416 and J24. Therefore, we only compared J2416 and 5237 to detect differentially expressed genes (DEGs).

**FIGURE 5:**
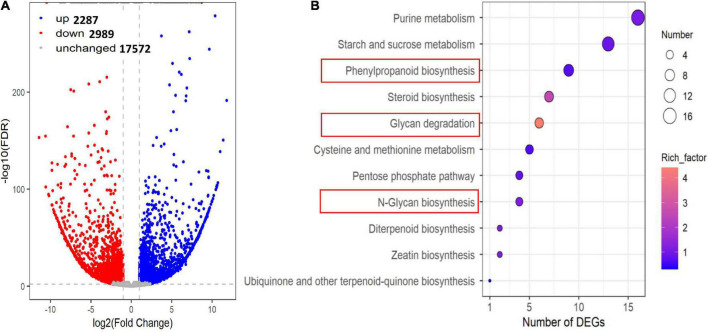
Differentially expressed genes (DEGs) between J2416 and 5237. **(A)** Volcano plot of DEGs. Blue and red dots represent significantly up- and down-regulated genes, respectively, in J2416 compared with 5237. **(B)** KEGG pathways enriched with up-regulated genes in J2416.

We detected 5276 DEGs between J2416 and 5237, of which 2287 (43.34%) were up-regulated in J2416 (Figure 5A). Among the up-regulated genes in J2416, 375 were annotated in 72 KEGG networks, 11 of which matched with the pathways of differentially accumulated metabolites (Figure 5B). The number of up-regulated genes in each of these KEGG pathways varied from 1 to 16. Among them, the phenylpropanoid pathway was related to lignin metabolism, and the glycan pathway was related to cellulose and hemicellulose metabolism. As we found that cellulose, hemicellulose and lignin were significantly enriched in J2416, and these substances were reported to be related to stalk strength, we focused on the genes related to these three substances. By searching the MAIZEWALL database and other reports (Penning et al., 2009; Wang et al., 2021), we listed 28 candidate genes which were related to cellulose, hemicellulose and were up regulated in J2416 and (Table 1).

**TABLE 1:** Up-regulated genes in J2416 related to synthesis of cellulose, hemicellulose, and lignin.

Trait	Gene_ID	Annotation	FPKM (5237)	FPKM (J2416)	log2(FC)	FDR	Refs
Cellulose	Zm00001d032188	Cellulose synthase	0.08	5.18	5.42	2.04E−53	Li et al., 2017
Cellulose	Zm00001d047276	COBRA-like protein 4(bk2)	12.99	95.8	2.88	2.79E−71	Sindhu et al., 2007
Cellulose	Zm00001d027525	Endochitinase B	8.09	30.8	1.86	1.32E−08	Kwon et al., 2007
Hemicellulose	Zm00001d053888	Glycosyltransferase	0	3.78	6.69	1.19E−28	Wu et al., 2010
Hemicellulose	Zm00001d046960	Glycosyltransferase	0	13.14	8.2	4.70E−51	Wu et al., 2010
Hemicellulose	Zm00001d023952	Glycosyltransferase	0	7.16	7.5	4.12E−40	Wu et al., 2010
Hemicellulose	Zm00001d047562	Glycosyltransferase family 47	0.57	1.56	1.36	1.86E−03	Zhang et al., 2011
Hemicellulose	Zm00001d036122	Glycosyltransferase family 61	1.46	4.28	1.4	6.52E−03	Chiniquy et al., 2012
Hemicellulose	Zm00001d008496	Glycosyltransferase family 61	0.12	1.79	3.32	4.70E−11	Chiniquy et al., 2012
Hemicellulose	Zm00001d028642	Glucuronosyltransferase	1.01	2.49	1.24	1.29E−03	Gao et al., 2020
Hemicellulose	Zm00001d008795	UDP-glycosyltransferase 74B1	0.23	1.1	2.02	2.13E−05	Penning et al., 2009
Hemicellulose	Zm00001d019256	UDP-glycosyltransferase 76C1	10.17	20.05	1	1.24E−10	Penning et al., 2009
Hemicellulose	Zm00001d034068	UDP-glycosyltransferase 83A1	0.27	1.42	1.99	1.14E−03	Penning et al., 2009
Hemicellulose	Zm00001d021755	UDP-glycosyltransferase 88A1	1.83	10.42	2.37	7.04E−10	Guo et al., 2021
Hemicellulose	Zm00001d021732	UDP-glycosyltransferase 88A1	0.04	0.94	3.64	1.37E−09	Guo et al., 2021
Hemicellulose	Zm00001d003102	UDP-glycosyltransferase 92A1	1.08	7.02	2.53	1.67E−09	Penning et al., 2009
Hemicellulose	Zm00001d003626	Xylem NAC domain	9.83	58.86	2.56	2.79E−33	Zhong et al., 2008
Hemicellulose	Zm00001d029814	Xyloglucan endotransglucosylase	1.95	6.67	1.74	4.95E−12	Xie et al., 2022
Hemicellulose	Zm00001d024382	Xyloglucan endotransglucosylase	0.48	1.74	1.72	4.19E−05	Xie et al., 2022
Lignin	Zm00001d020402	Cinnamyl alcohol dehydrogenase 6(CAD6)	36.4	78.31	1.12	4.24E−12	Halpin et al., 1998
Lignin	Zm00001d034015	Exoglucanase1(exg1)	14.2	22.7	1.16	2.52E−15	Furukawa et al., 2014
Lignin	Zm00001d039837	Hydroxy cinnamoyl transferase	12.76	27.49	1.15	3.65E−27	Serrani-Yarce et al., 2021
Lignin	Zm00001d025140	Hydroxy cinnamoyl transferase	0.03	2.2	4.25	1.40E−10	Serrani-Yarce et al., 2021
Lignin	Zm00001d020530	Hydroxy cinnamoyl transferase11	25.24	56.43	1.19	1.53E−17	Serrani-Yarce et al., 2021
Lignin	Zm00001d035055	Peroxidase 53	10.74	47.44	2.15	1.06E−45	Marjamaa et al., 2009
Lignin	Zm00001d047984	Peroxidase 53	0.13	3.08	3.57	1.4E−12	Marjamaa et al., 2009
Lignin	Zm00001d003016	Phenylalanine ammonia lyase2(PAL2)	13.95	61.03	2.15	4.7E−113	Rohde et al., 2004
Lignin	Zm00001d029391	Phospho-2-dehydro 3 deoxyheptonate aldolase 2 (PDA2)	5.34	13.43	1.32	2.3E−12	Schomburg and Salzmann, 1990

Three of the up-regulated genes in J2416 were related to cellulose content, and their -fold change values in J2416 ranged from 1.86 to 5.42. The genes were found to encode cellulose synthase, chitinase, and COBRA-like protein 4 (*bk2*), which was first identified from a brittle stalk mutant (Sindhu et al., 2007). The functions of other genes were related to cellulose synthesis and stalk lodging resistance (Table 1).

Sixteen of the up-regulated genes in J2416 were related to hemicellulose, and their fold change values ranged from 1 to 8.2. These genes encoded glycosyltransferase family members, UDP-glycosyltransferase, and xyloglucan endotransglucosylase. Zm00001d021755 encoding UDP-glycosyltransferase 88A1 was previously identified from a comparison of lodging-resistant and lodging-sensitive accessions (Guo et al., 2021). The other genes encoded products with roles in hemicellulose synthesis or stalk lodging resistance (Table 1).

The phenylpropanoid pathway which involved in the biosynthesis and degradation of lignin contained nine up-regulated genes with their fold change values in J2416 ranging from 1.19 to 4.25 (Table 1). These genes encoded multiple key enzymes in lignin metabolism, such as phenylalanine ammonia lyase2 (PAL2), cinnamyl alcohol dehydrogenase 6 (CAD6), peroxidase 53, and hydroxy cinnamoyl transferase (Table 1).

**FIGURE 6:**
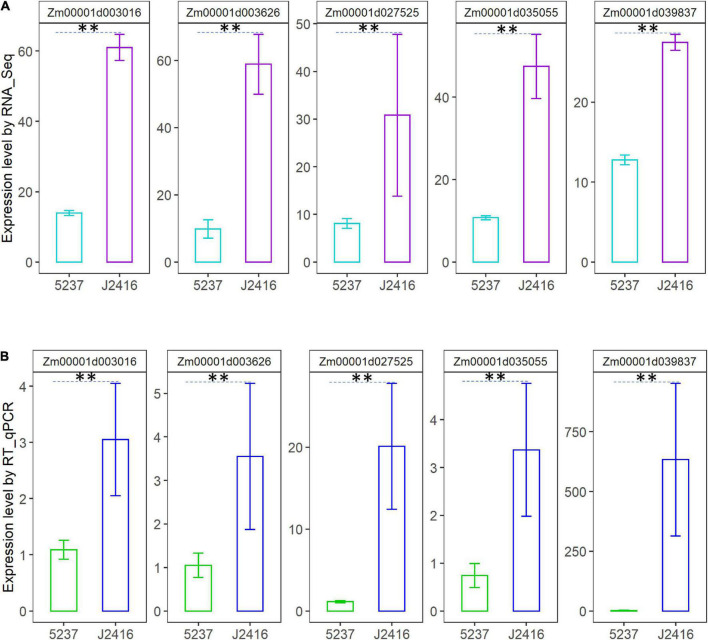
Expression levels of five randomly selected genes related to cellulose, hemicellulose and lignin in J2416 and 5237. **(A)** Is the expression level detected by RNA_Seq. **(B)** Is the expression level detected by RT-qPCR. **Means the significant difference at 0.01 level.

To validate the expression profiles of the genes in Table 1, five DEGs were randomly selected for RT-qPCR (Figure 6). From the results of RT-qPCR of these genes, we can see the expression level of J2416 were significantly higher than that of 5237. The concordance between RT-qPCR and RNA-Seq results confirmed that the findings from RNA-Seq were credible.

## Discussion

### The stalk strength characteristics of J2416 were inherited from J24

The field practice showed that J2416 and many cultivars bred of J2416 have good lodging resistance. In order to reveal the reason of the excellent stalk strength of J2416, we analyzed the stalk strength and related traits of J2416 and its parents (J24 and 5237). The results showed that the stalk strength of J2416 was significantly greater than that of 5237 in multiple environments, but just slightly higher than J24. Differential accumulated metabolites were also very few between J2416 and J24. The main reason was the bred process and the genetic background. From the pedigree, J2416 was bred from the cross of J24 and 5237 and the F1 backcrossed with J24 (Figure 1). The proportions of J2416 retained nearly 80.96% and 19.04% of identity by descend (IBD) segments from J24 and 5237, respectively, suggested the J24 had a greater genetic contribution to J2416 (Zhao et al., 2020b). Therefore, we hold the view that the stalk strength characteristics of J2416 inherited and better than the characteristics of J24.

### Important roles of cellulose, hemicellulose, and lignin in stalk strength

Stalk strength is a mechanical characteristic that results from the contributions of multiple factors, including the internal structure of stalk and its chemical composition. In this study, we analyzed the inbred line J2416 with excellent stalk strength and its parents to determine which phenotypic, biochemical, and genetic factors are related to stalk strength.

We found that the stalk strength of J2416 was positively correlated with the rind thickness and sclerenchyma cell thickness. The main components of the cell wall are cellulose, hemicellulose, and lignin, so we quantified each of these components in the stalks of the three inbred lines. The contents of cellulose, hemicellulose, and lignin in J2416 were slightly higher than those in J24 and significantly higher than those in 5237. This result was consistent with the analyses of stalk mechanical strength and stalk anatomic structure, indicating that these three substances play vital roles in the stalk strength of J2416. Many studies have detected strong correlations between the mechanical strength of the stalk and the contents of cellulose, hemicellulose, and lignin (Vignols et al., 1995; Halpin et al., 1998; Sindhu et al., 2007; Tang et al., 2014; Li et al., 2015; Xiong et al., 2019). For example, the weaker stalk strength of the maize brittle stalk mutant *bk2* was found to be due to lower cellulose and hemicellulose contents resulting from a mutation in a cellulose-related gene (Sindhu et al., 2007). The brown midrib mutants *bm1*–*bm5* were found to have mutations in genes encoding lignin biosynthetic enzymes, leading to decreased contents of total lignin or lignin monomers, or a change in the ratio of monomers, resulting in reduced stalk strength (Vignols et al., 1995; Halpin et al., 1998; Tang et al., 2014; Li et al., 2015; Xiong et al., 2019).

### Metabolites and candidate genes related to excellent stalk strength

Previous studies have shown that genes and metabolites related to the phenylpropanoid pathway were expressed at significantly lower levels at the fractured position of the stalk than in other parts of the stalk (Wang et al., 2019). The phenylpropanoid pathway is responsible for lignin synthesis. This pathway begins with phenylalanine, which serves as the substrate for the synthesis of lignin monomers *via* the actions of phenylalanine ammonia lyase (PAL), cinnamic acid 4-hydroxylase (C4H), and 4-coumarate-coenzyme A ligase (4CL) (Weng and Chapple, 2010). In this study, we found that the phenylpropanoid pathway, followed by the glycan synthesis and metabolism pathways, were most enriched with up-regulated metabolites in J2416.

To identify genes related to the control of phenylpropanoid metabolism, we detected DEGs between J2416 and 5237 by analyzing transcriptomic data. Nine genes in the phenylpropanoid pathway were up-regulated in J2416, encoding enzymes including PAL2, CAD6, and peroxidase. The first step in the phenylpropanoid pathway is catalyzed by PAL (Yoon et al., 2015). Another study found that the lignin content in the *pal1 pal2* double mutant was reduced in parallel with an increase in the ratio of the S unit to G unit of lignin monolignol (Rohde et al., 2004). The last step of monolignol biosynthesis is catalyzed by CAD, which converts hydroxyl-cinnamaldehydes into their corresponding alcohols (Hirano et al., 2012). Analyses of the midrib mutant *bm1* revealed a mutation in the *CAD* gene that resulted in severely reduced CAD activity, which affected both the total amount of lignin and the structure of lignin monomers (Halpin et al., 1998). The main function of peroxidase is to oxidize or dehydrogenate free lignin monomers and couple them to form lignin polymers (Marjamaa et al., 2009).

Three genes related to cellulose and 16 related to hemicellulose were among the genes that were up-regulated in J2416 compared with 5237. *Bk2* was first identified in a maize brittle stalk mutant, in which the mutation resulted in a 40% reduction in total cellulose (Sindhu et al., 2007). Cellulose synthase catalyzes the polymerization of UDP-glucose to form glucan chains. Mutations in this type of gene in *Arabidopsis* led to defects in cellulose synthesis (Zhang et al., 2011). Chitin is a high molecular weight polysaccharide polymer. Another maize brittle stalk mutant, *bk4*, was found to be due to the mutation of a gene encoding a chitinase-like protein, and resulted in significantly decreased p-coumaric acid, glucose, mannose, and cellulose contents in the stalk (Jiao et al., 2019). The 16 hemicellulose-like genes family can be found in MAIZEWALL database (Penning et al., 2009). Especially, the gene Zm00001d021755 encoding UDP-glycosyltransferase 88A1was also identified in another research with high lodging resistance (Guo et al., 2021). The genes identified in this study can be as the candidate genes related to stalk strength.

## Conclusion

Three maize inbred lines with pedigree relationships but significant differences in stalk strength were analyzed to identify traits related to superior stalk strength at the cellular, physiological, metabolic and transcript levels. Our analyses revealed nine genes related to lignin content, three related to cellulose content, and sixteen related to hemicellulose content that were up-regulated in the line with the strongest stalks. High transcript levels of these genes in the line with excellent stalk strength resulted in increased total amounts of related metabolites and macromolecular polymers and thicker cell walls, resulting in a stiff stalk.

## Data availability statement

The datasets presented in this study can be found in online repositories. The names of the repository/repositories and accession number(s) can be found below: https://www.ncbi.nlm.nih.gov/, PRJNA843174.

## Author contributions

XW and JZ conceived and designed the experiments. YC, XS, JL, and RZ carried out the experiments. XW, YC, WS, and RW analyzed the data. XW wrote the manuscript. YJ revised the manuscript. All authors have read and agreed to the published version of the manuscript.

## Footnotes

1
http://www.kegg.jp/kegg/pathway.html
