# Supplementary material for: Characteristics and candidate genes associated with excellent stalk strength in maize (Zea mays L.)
Source: Front Plant Sci. 2022 Jul 28;13:957566. doi: 10.3389/fpls.2022.957566 (PMC9367994; doi:10.3389/fpls.2022.957566)
Supplement: Supplementary file 1 [file Table_1.docx]

**Supplementary Table 1 Primers used for RT-qPCR**

| Genes | Forward Primer | Reverse Primer |
| --- | --- | --- |
| Zm00001d003016 | ACCACTCAACTCACCAACCC | GATGATGCTGATGCGGTGTG |
| Zm00001d003626 | ATATCCTTTGCACCTGCGCT | TGACGGGAAGAAGTGGAAGC |
| Zm00001d027525 | ATCAAAGCAAGCCATGTGGAG | GGCGTCGTAGGTGTAGAAGG |
| Zm00001d035055 | ATGGTGAGATCAGGCACACG | ATCTTGGTTTGCATGGGGCA |
| Zm00001d039837 | GTCGCCACACTGTTTTCAGG | ACAAAGACTCGCACGATTGC |
| ZmActin | GCTATCCAGGCTGTTCTTTCG | CATTAGGTGGTCGGTGAGGT |
